# Response Surface Methodology to Explore the Influence Mechanism of Fiber Diameter in a New Multi-Needle Electrospinning Spinneret

**DOI:** 10.3390/polym16152222

**Published:** 2024-08-05

**Authors:** Jianmin Jiang, Xiaojie Chen, Han Wang, Weicheng Ou, Jiayi He, Maolin Liu, Zehui Lu, Jingyi Hu, Gaofeng Zheng, Dezhi Wu

**Affiliations:** 1 State Key Laboratory of Precision Electronic Manufacturing Technology and Equipment, Guangdong University of Technology, Guangzhou 510006, China; 17301991036@163.com (J.J.); wanghangood@gdut.edu.cn (H.W.); 2111901292@mail2.gdut.edu.cn (W.O.); addone0801@163.com (J.H.); liumaolingdut2020@163.com (M.L.); 13268638338@163.com (Z.L.); 13632225738@163.com (J.H.); 2 School of Electromechanical Engineering, Guangdong University of Technology, Guangzhou 510006, China; 3 Pen-Tung Sah Institute of Micro-Nano Science and Technology, Xiamen University, Xiamen 361102, China; zheng_gf@xmu.edu.cn (G.Z.); wdz@xmu.edu.cn (D.W.)

**Keywords:** multi-needle electrospinning, spinneret, simulation, response surface method

## Abstract

Multi-needle electrospinning is an efficient method for producing nanofiber membranes. However, fluctuations in the fluid flow rate during the process affect membrane quality and cause instability, an issue that remains unresolved. To address this, a multi-stage flow runner spinneret needs to be developed for large-scale nanofiber membrane production. This paper uses COMSOL finite element software to simulate polymer flow in the spinneret runner. From this, the velocity field distribution and velocity instability coefficient were obtained, providing theoretical guidance for optimal spinneret design. In addition, response surface analysis (RSM) was used to experimentally explore the process parameters, and then residual probability plots were used for reliability verification to evaluate the effect of each process parameter on fiber diameter. These process parameters can guide the controlled production of nanofibers during multi-needle electrospinning.

## 1. Introduction

Electrospinning is the primary technology for nanofiber membrane fabrication, enabling the continuous production of nanoscale fibers from various polymers with applications in environmental protection, biomedicine, and microelectronics [1,2,3,4]. Single-nozzle electrospinning, despite its simplicity, faces productivity challenges [5]. To address this, multi-needle and needleless electrospinning techniques have emerged for efficient, industrial-scale production [6,7]. Needleless electrospinning uses rotating or stationary spinnerets to generate polymer jets, avoiding needle clogging [8,9]. However, it has limitations in productivity, maintenance costs, and fiber uniformity due to uneven electric field distribution [10]. Multi-needle electrospinning offers advantages in material adaptability and application diversity [11,12,13], improving productivity and fiber diameter control as needle numbers increase.

Multi-needle electrospinning devices comprise a high-voltage power supply, solution supply, multi-needle spinning module, and collector [14]. Key design parameters include applied voltage [15], flow rate [16], and needle length-to-diameter ratio [17]. Research has shown that applied voltage and flow rate significantly affect the process. Angammana et al. [18] demonstrated that stronger electric field interference in multi-needle systems increases deflection angles and reduces fiber uniformity. Zhou et al. [19] found that interference strength correlates with needle tip pitch, with smaller pitches leading to stronger interference and lower fiber quality. While most research focuses on needle configurations, few studies address flow rate control, which is crucial for efficiency and productivity in electrospinning [20,21].

Maintaining uniform liquid flow for each needle tip in multi-needle electrospinning is crucial for achieving consistent nanofiber diameter and quality. As the number of needles increases, precise flow control becomes challenging [22,23]. Inconsistent flow rates lead to uneven electric field distribution, resulting in non-uniform fibers. Optimizing the spinneret design, particularly the flow runner layout, is essential for synchronized flow distribution [24]. Kim et al. demonstrated through computational fluid dynamics that a hierarchical design with bend headers can synchronize flow in multi-needle systems [25]. While cylindrical spinnerets with linearly arranged needles enhance productivity, they often struggle to ensure consistent flow distribution [26]. Simulations have shown a strong correlation between spinneret runner structure and flow velocity/pressure distribution [27]. While hanger-type runners have demonstrated uniform flow distribution in melt blowing, their application in multi-needle electrospinning remains unexplored [28]. Overall, developing a well-designed spinneret that guarantees uniform flow is paramount for advancing large-scale, high-quality nanofiber fabrication.

Previous studies have addressed some of the problems in multi-needle electrospinning, but electrostatic spinning has a very large difference in diameter between industrial production and laboratory studies, so a more systematic study of this is needed. In order to increase the productivity of nanofibers, by combining the hanger-type die heads’ runners [29] and the research of spinneret runners [30,31], this paper proposes a novel multi-stage runner spinneret, which satisfies requirements for uniform distribution and velocity consistency of the flow. First, it utilizes simulation to analyze outlet velocity distributions across runners. Afterwards, the spinneret runner geometry is optimized, ensuring a smooth delivery of pressure or flow rate to each needle tip. And then, the process parameter effects on nanofiber diameter is explored by constructing a regression model with response surface methodology (RSM). Finally, the stability of the nanofiber production is improved. Results show that the spinneret is capable of stable fabrication with multiple jets and has great potential in the industrial production of nanofibers.

## 2. Design and Simulation of Spinneret

The stability of the polymer outflow in the spinneret and the uniformity of the flow distribution are the keys to determining the quality of the nanofiber membrane. To ensure the performance of multi-needle electrospinning, the new multi-stage flow runner spinneret was designed. Finite element analysis was used to simulate the velocity during the polymer flow under different flow runner angles, and then the influence of flow runner with different structures on the stability of polymer flow was analyzed, which provided guidance for the optimization of the spinneret.

**Figure 1:**
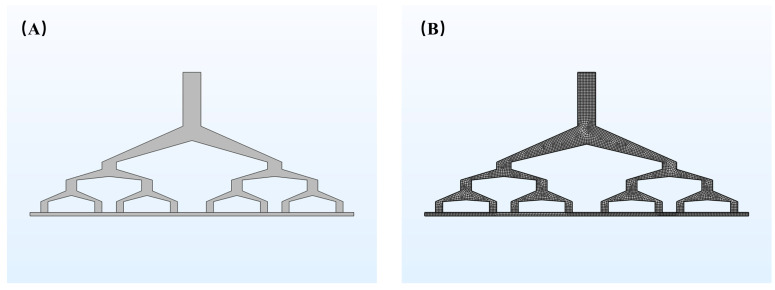
(**A**) Channel model of the spinneret device. (**B**) Mesh generation.

The geometric model and mesh division of the designed spinneret is shown in Figure 1A,B, respectively. The runners directly determine whether the polymer flow can be even, so it is very important to consider the internal runner structure of the spinneret. The structure of the multi-stage runners roughly resembles a clothes hanger, as shown in Figure 1A, which can avoid the phenomenon of superfast flow and an uneven distribution of the polymer in the vertical flow runners. The polymer is squeezed into the flow runner inlet by applying high pressure. The polymer after entering the entrance is divided into two parts through the primary flow runner, and then the two parts are further divided into four parts through the secondary flow channel, and then divided into four more parts through the third flow channel, until finally the parts reach each outlet of the spinneret. The abovementioned structure facilitates a uniform polymer flow from the entrance to each outlet. The design of the new spinneret is simplified to eight outlets for simulation. The width is designed to be 800 mm, while the height is 240 mm. The inlet size of the flow runner is 40 mm × 80 mm and the inlet size of the secondary and third flow runners are 25 mm × 15 mm. The inlet size of the runners is 20 mm × 20 mm and the height of the polymer retention area is 15 mm. After the geometric model is established, the meshing is the key to simulation. Considering the requirements of meshing, some simplifications are made to the geometry of the flow runners, and small excessive fillets are ignored to improve calculation precision and speed. COMSOL is used to simulate the isothermal polymer flow process during electrospinning. The characteristics of polymer flow are mainly high viscosity and low Reynolds number. In order to meet the engineering conditions and simplify the calculation, the following necessary assumptions are made for the polymer flow. Steady-state flow thus occurs in the flow runners, where the wall velocity is set to zero by only considering the viscous force.

**Figure 2:**
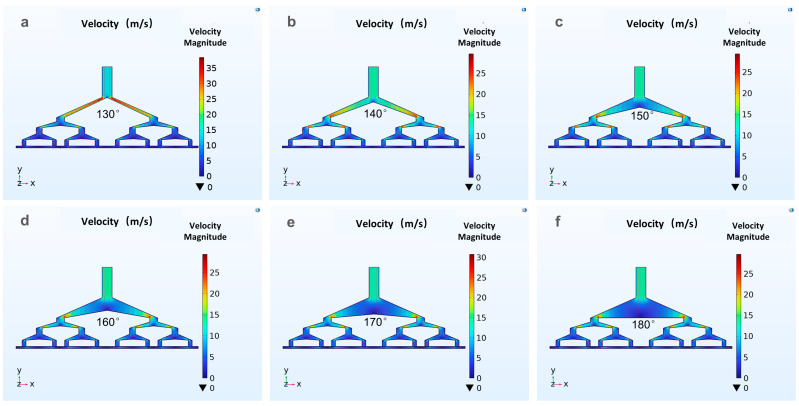
Polymer velocity distribution at different angles of spinnerets: (**a**) 130°, (**b**) 140°, (**c**) 150°, (**d**) 160°, (**e**) 170° and (**f**) 180°.

The results of the finite element analysis can guide the geometry and evaluation of different structural runner configurations for the novel spinneret. Either a two-dimensional or three-dimensional flow-field velocity distribution can be easily attained. Figure 2a–f show the flow velocity distribution of the polymer at different runner angles. Six kinds of different spinneret flow runner structures are established to study the influence of different angles on the velocity field and calculate the velocity distribution at the outlet. The differences among the above six structures are the angles between them and the primary flow runner, which are 130°, 140°, 150°, 160°, 170°, and 180°, respectively. A gear pump is selected to continuously supply the polymer, and the inlet pressure is 0.5 Mpa. The research results have guidance significance for the optimization of new spinnerets.

**Figure 3:**
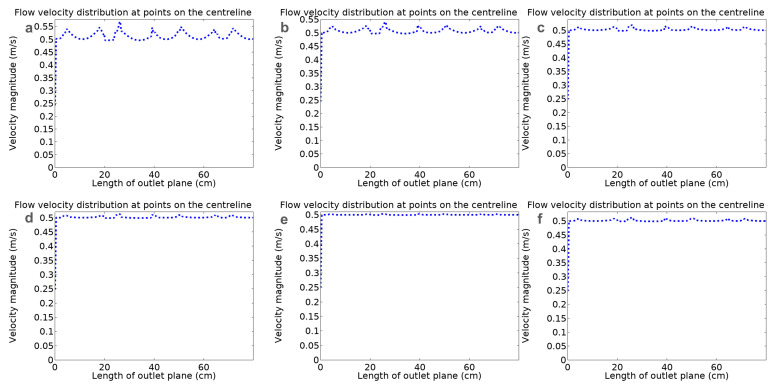
Velocity distributions of polymer solution through novel spinneret channels with different angles: (**a**) 130°; (**b**) 140°; (**c**) 150°; (**d**) 160°; (**e**) 170° and (**f**) 180°.

According to the flow distribution of the polymer at different angles in the spinneret runner, the flow velocity distribution curve at the outlet can be obtained, as shown in Figure 3a–f. The abscissa is the length of the outlet and the ordinate is the flow velocity at different parts of the outlet. It can be seen that each curve represents the velocity at the outlet, and the velocity distribution at each point of the outlet is drawn. In order to compare the flow rate at the same outlet with different angles, 100 points are taken evenly from the horizontal direction, and this along with the flow rate of the polymer vertical to the outlet plane are extracted for analysis. U is set as the maximum deviation between the real flow velocity of each point and the average flow velocity, which is an indicator for evaluating the non-uniformity degree of the flow velocity. The $V_{i}$ (I = 1, 2, 3, 4, …, 100) represents the polymer velocity at each point in the direction vertical to the outlet plane.

(1) $$U=\frac{\left|V_{i}-\frac{1}{100}\sum V_{i}\right|}{\frac{1}{100}\sum V_{i}}\times 100\%$$

**Figure 4:**
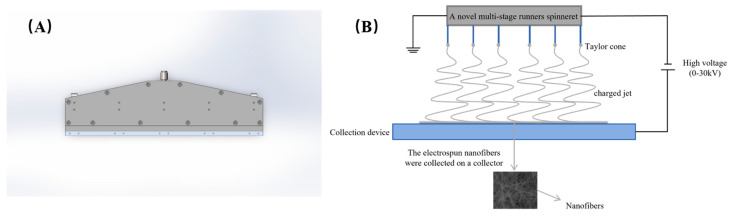
(**A**) Schematic diagram of multi-needle electrospinning setup and principle. (**B**) Structural diagram of the novel spinneret plate.

Since different flow runners meet and merge in the polymer retention area, more fluid will flow longitudinally, i.e., vertical to the direction of the outlet plane, which results in the formation of an uneven velocity area. The increase in the angle will slow down the polymer flow rate, thereby making the outlet velocity more uniform. After passing through the primary flow runner, the velocity gradually slows down. Before entering the secondary flow runner, the polymer flow generates fluctuation. This is because the size of the secondary flow changed prior to entering the runner, which caused a readjustment of the fluid flow. Finally, after passing the third flow runner, the velocity in the spinneret decreases. Each flow runner shrinks by a certain proportion until it connects in the flow retention area, which all play the role of distribution and buffering. The primary runner affects the polymer distribution and determines the polymer supply situation of the subsequent runners. It can be seen from Figure 4 that as the angle increases, the flow velocity at the outlet centerline becomes more and more uniform. This trend can be seen from the calculation results of the non-uniformity index, *U*. When the angle is 130°, *U* is 9.1%. When the angle increases to 140°, *U* is 8.36%. When the angle is 150°, *U* is 7.81%. When the angle is 160°, *U* is 5.67%. When the angle is 170°, *U* is 4% and when the angle is 180°, *U* is 6.38%. Through simulation, it was found that as the angle increases, the uniformity of the overall outlet flow rate can be further improved. However, when the angle is 180°, the polymer flow velocity slows down in the area, which also affects the uniformity of flow distribution. It is therefore easy to produce vortex and flow buffering effects at the end of the primary runner, affecting the uniformity of flow distribution.

## 3. Experiment

### 3.1. Experimental Materials

Polyvinylidene fluoride (PVDF) with an average molecular weight of 300,000 g/mol (St. Louis, MA, USA, Sigma-Aldrich) and N, N-dimethylformamide, called DMF (Shanghai, China, Shanghai Aladdin Chemical Co.), were used as received without further purification.

### 3.2. Characterization

In order to observe the fiber morphology more clearly, the following instruments were used to assist in characterization. The plasma-thin-film sputtering instrument (IBS, ISC150, China) was used to spray gold onto the fiber membrane. Then, the scanning electron microscope (SEM, TM3030, Hitachi) was used to observe the morphology of the nanofibers. Next, the nanofiber diameter was measured using ImageJ software (ImageJ 1.54g) wherein approximately 100 nanofibers in each sample were selected to measure the diameter. Finally, the Origin software(Origin 2021) was used to draw the distribution frequency of the fiber diameter.

### 3.3. Experimental Design

A novel multi-stage runner spinneret was used to fabricate the nanofiber membranes in this experiment, as shown in Figure 4A. First, the prepared polymer solutions are transported to the liquid supply module. And then, the distance between the collector and the needles was adjusted to make sure that the distance was suitable. Next, the high spinning voltage was applied in the spinneret by a high-voltage power module. Under the influence of the electric field force, the jet was pulled to the grounded collector, as shown in Figure 4B. Finally, the nanofibers composed of micro–nano-sized jets were obtained and gradually generated the membrane products.

In order to study the influence between process parameters and diameter on the generated nanofibers, the Box–Behnken Design (BBD) response surface method was used to design experimental plans and analyze the experimental results. The floating range of the process parameters was found based on single-factor experiments individually, which were needed to optimize and divide them into three factor levels as shown in Table 1. The coding levels of the spinning voltage were 55 kv, 60 kv and 65 kv; the collection distances were 20 cm, 25 cm and 30 cm; and the solution concentrations were 11 wt%, 13 wt% and 15 wt%. According to the BBD method, the 17 sets of coding factors of the process parameters were combined to design optimal experiments. After that, every nanofiber diameter datum was obtained, as shown in Table 2.

## 4. Results and Discussion

### 4.1. RSM Testing

Establishing a regression model can optimize process parameters and predict response indicators, and finally obtain the optimal regression fitting equation. Using Design-Expert software (Design-Expert 11.0), a response surface regression model and a response index mathematical model were constructed for the average fiber diameter data obtained from the experiment, as shown in Table 3. Square sum represents the portion of variation explained by the model and reflects the strength of the relationship between the factor and the response variable. In the analysis of variance, these square sums were used to calculate the mean square, which was then used for significance testing. The model F-value of 60.50 implied that the model was significant. Comparing and evaluating the *p*-value of the above model with the significance level, the *p*-value was used to analyze the significance of the objected model. *p* > 0.1 indicates that the response model is not significant. When *p* < 0.05, it means that the model is at a significant level. When *p* < 0.0001, it means that the response model has reached an extremely significant level. The analysis results show that C and C2 are important model factors, and their *p* values are less than 0.0001, which indicates that the effect of solution concentration is extremely significant. The *p* value of B is less than 0.05, which indicates that the collection distance has a significant effect on the nanofiber diameter. The *p* values of the remaining factors are all higher than 0.1, which indicates that they have a slight effect on the nanofiber diameter. Comparing the mean square values, it is found that the influence strength of process parameters on the nanofiber diameter is C > B > A, and the strength of interaction is AB > BC > AC. In this experiment, the response index mathematical model established using the RSM method is

(2) $$\begin{array}{l} Y/\mathrm{um}=0.208-0.06A/\mathrm{kV}+0.11B/cm+0.08C/wt\%-0.01AB/(kV\times cm)-\\ 0.02AC/(kV\times wt\%)-0.02C/wt\%-0.04A^{2}/(kV\times kV)+0.06B^{2}/(cm\times cm)+\\ 0.08C^{2}/(wt\%\times wt\%) \end{array}$$

where Y is the diameter of the PVDF nanofiber membrane, and the unit of *Y* is um.

**Figure 5:**
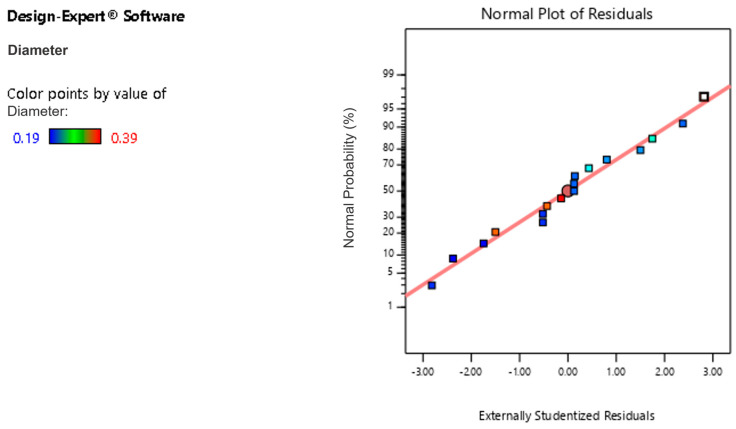
The residual of the average nanofiber diameter.

The residual normal probability plot is used to verify the model reliability by observing the data to see whether they essentially obey a linear distribution. The normal probability plot is shown in Figure 5. The X-axis of Figure 5 represents the theoretical quantiles of the standard normal distribution. The Y-axis represents the cumulative probability of the data points under the standard normal distribution. The data in Figure 5 are ultimately shown as a beeline, which indicates that the accuracy of the model is reliable.

### 4.2. Response Surface Analysis

**Figure 6:**
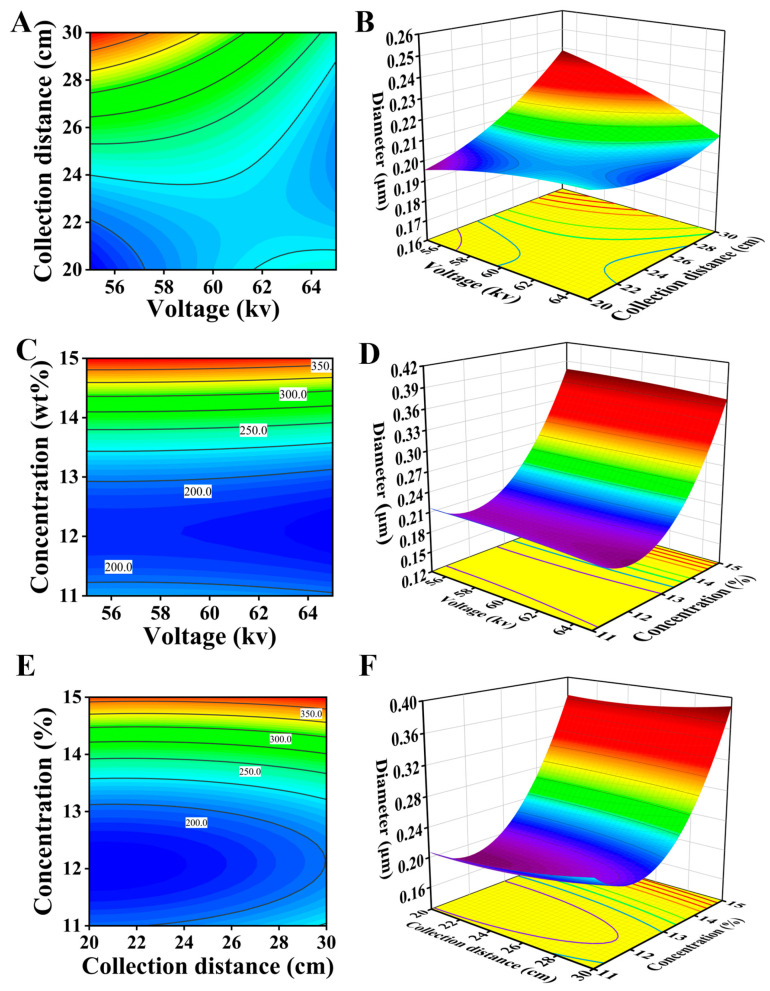
(**A**) vltage and collection distance Contour Plots; (**B**) Voltage and collection distance response surface plots; (**C**) Voltage and Concentration Contour Plots; (**D**) Voltage and Concentration response surface plots; (**E**) Collection Distance and Concentration Contour Plots; (**F**) Collection Distance and Concentration response surface plots.

The influence of different process parameters on the average diameter of nanofibers was studied, and then the response surface curve was drawn using the Design-Expert software(Design-Expert 11.0), as shown in Figure 6. The process parameters are voltage, collection distance, and solution concentration. It shows the 2D and 3D response surface profiles of the interaction between voltage and collection distance in Figure 6A,B. As the spinning voltage increases, the average diameter of the nanofibers relatively decreases. Because the electric field intensity and the repulsive force of the fluid jet increase as the voltage increases, it is conducive to fiber thinning, and the average nanofiber diameter will directly decrease. However, a higher spinning voltage will also aggravate the electric field interference, which affects the stable formation of Taylor cones and the jet flow trajectory. The above phenomenon is not conducive to fiber thinning, so it can be seen that the influence of higher voltage in the nanofiber diameter is very weak. Figure 6C,D show the 2D and 3D response surface profiles of the interaction between voltage and solution concentration. From the profiles, it can be described that the solution concentration has a significant effect on the average nanofiber diameter. When the concentration is low (about 11–12%), the increasing concentration will improve the viscosity, which is conducive to the generation of better fibers. When the concentration continues to increase (higher than 12%), the viscosity increases rapidly, and it becomes more difficult for the electric field force to exceed the surface tension of the jet; hence, the diameter becomes larger. Figure 6E,F show the 2D and 3D response surface profiles of the interaction between collection distance and solution concentration. The value of average nanofiber diameter increases as the collection distance value increases. When the collection distance is farther, the flight time of the jet is longer, and the solvent fully evaporates, thereby improving the concentration. The high-concentration solutions have a lower probability to generate larger-diameter fibers. Additionally, the long-distance flight time of the jet will also attenuate the electrostatic potential, which weakens the electric field force and ultimately affects the fiber diameter.

### 4.3. Optimization and Validation

**Figure 7:**
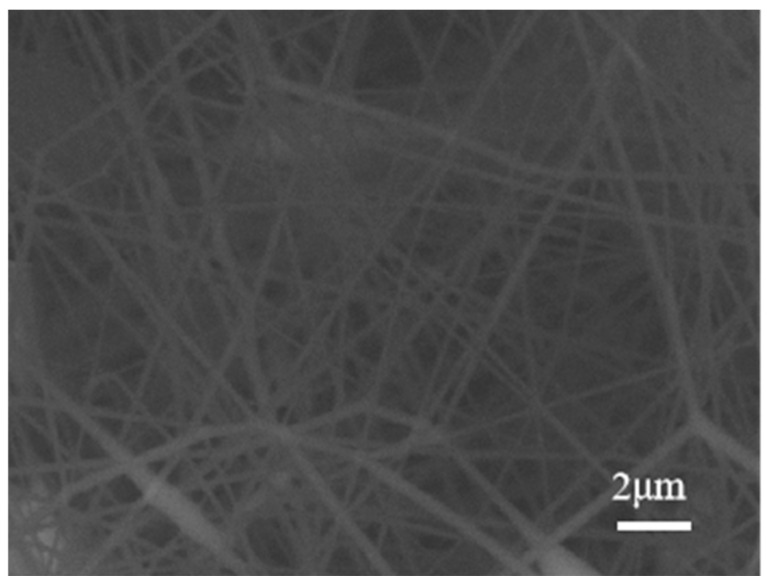
SEM images of experimental samples.

The process parameter equation from the RSM model was analyzed in the Design-Expert software (Design-Expert 11.0), and the extreme points of the response indicators were automatically explored through the optimization, which allowed for the determination of the best process parameter configuration. The parameters were configured as 12% of solution concentration, 25 cm of the collection distance and 59 kV of the spinning voltage. The average value of the nanofiber diameter that was used as the best parameter configuration was 0.199μm. This parameter configuration was used to conduct the repeated experiments to verify the optimal results, and the average diameter used in the repeated experiments was 0.195 μm. The SEM images are shown in Figure 7. Compared with the optimal results, 0.199 μm, the deviation was only 2.11%. This indicates that the regression equation constructed through RSM was relatively accurate and can be used for the process optimization of multi-needle electrospinning systems.

### 4.4. Microscopic Morphology

**Figure 8:**
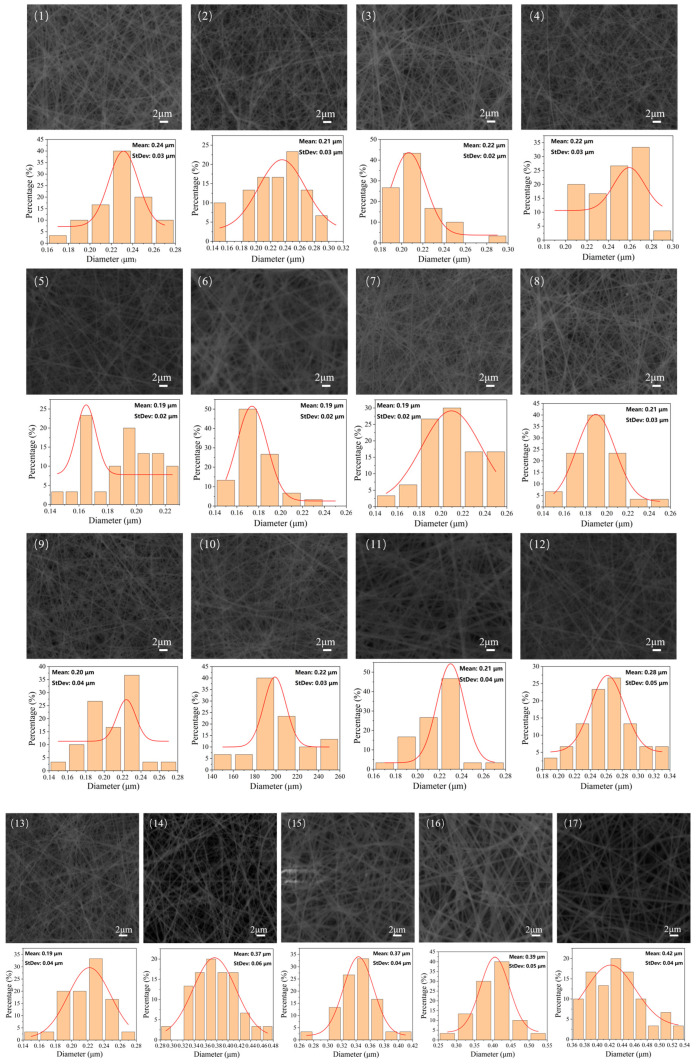
(1)–(4) are electron micrographs and diameter distributions for 11% concentration; (5)–(13) are electron micrographs and diameter distributions for 13% concentration; (14)–(17) are electron micrographs and diameter distributions for 15% concentration.

Scanning electron microscopy (SEM) is used to observe the microscopic morphology of the nanofiber membrane. ImageJ software(ImageJ 1.54g) is used to analyze the SEM images, in which approximately 100 nanofibers in each sample are selected to measure the diameter, and Origin software (Origin 2021) is used for drawing the distribution of fiber diameters. The SEM images and diameter distribution are shown in Figure 8. There are four groups with a concentration of 11 wt%, and its corresponding SEM images and fiber diameter distribution are displayed in Figure 8(1)–(4). Due to the relatively low concentration (11 wt%), bead-like nanofibers were formed. There are differences between the nanofiber diameters in the above four experimental results, and the average diameter of nanofibers is 0.24 μm. By comparing the nanofiber diameters in Figure 8(1) and (4), we can determine that the nanofiber diameter increases as the collection distance increases under the same 11 wt% concentration. By increasing the spinning voltage, the electric field intensity and the electric field force increase, which causes the polymer solution to suffer a greater tensile force during the spinning process as well as fabricates thinner nanofibers, (Figure 8(10)–(11)). As the voltage increases, thinner nanofibers can be obtained, while the difference in fiber diameter is reduced and the diameter is more uniform. There are nine groups of experimental results which were conducted with 13 wt% concentration, as show in Figure 8(5)–(13). Observing the SEM images, the overall morphology of the fiber membrane is evenly distributed and the diameters are relatively thin. The diameter range of the nanofibers is [0.20, 0.23] μm. There are four groups of experimental results which were conducted with 15 wt% concentration, as show in Figure 8(14)–(17). The nanofibers’ average diameter of the above four groups is 0.34 μm. Compared with the other experimental results involving low concentrations, the nanofibers fabricated with high concentration are thicker.

## 5. Conclusions

In this study, a novel multi-stage runner spinneret was designed to facilitate a large-scale fabrication of nanofiber membranes. COMSOL Multiphysics 6.0 was employed to simulate the velocity distribution of a polymer solution at various runner angles within the spinneret. The simulation results indicated that the non-uniformity (U) at the spinneret outlet was minimized when the primary flow channel angle was set to 170°. Electrospinning experiments were conducted using the Box–Behnken Design (BBD) response surface methodology for data analysis. A mathematical regression model was established to correlate the process parameters with the average fiber diameter. Subsequent significance and error analyses were performed on the model. The results revealed that solution concentration exerted the most significant influence on fiber diameter compared to voltage and collection distance. Analysis of the experimental results demonstrated that bead-shaped nanofibers were produced at a solution concentration of 11 wt%. At 13 wt% concentration, the nanofibers exhibited generally smaller diameters with the lowest standard deviation, indicating a uniform overall diameter distribution. The largest average diameters, ranging from 0.30 to 0.44 μm, were observed at a solution concentration of 15 wt%. Based on these findings, the optimal electrospinning process parameters were determined to be a PVDF concentration of 12 wt%, a collection distance of 25 cm and a spinning voltage of 59 kV. This study provides a convenient and effective approach for fabricating nanofibers with highly uniform diameters, contributing to the advancement of large-scale nanofiber membrane production.

## Figures and Tables

**Table 1:** Coding levels of electrospinning process parameters.

Design Variable	Denotations	Factor Level		
		−1	0	1
Voltage (Kv)	A	55	60	65
Collection distance (cm)	B	20	25	30
Concentration (wt%)	C	11	13	15

**Table 2:** Experimental schemes and results.

Experimental Coding	Working Voltage A (Kv)	Collection Distance B (cm)	Solution Concentration C (wt%)	Nanofiber Average Diameter (μm)
1	60	30	11	0.24 ± 0.03
2	60	25	13	0.21 ± 0.03
3	65	25	11	0.22 ± 0.02
4	60	25	13	0.22 ± 0.03
5	55	25	15	0.37 ± 0.02
6	65	30	13	0.19 ± 0.02
7	55	20	13	0.20 ± 0.02
8	60	25	13	0.21 ± 0.03
9	60	25	13	0.19 ± 0.04
10	60	25	13	0.20 ± 0.03
11	60	20	11	0.21 ± 0.04
12	60	30	15	0.42 ± 0.05
13	60	20	15	0.37 ± 0.04
14	65	20	13	0.19 ± 0.06
15	55	30	13	0.28 ± 0.04
16	55	25	11	0.22 ± 0.05
17	65	25	15	0.39 ± 0.04

**Table 3:** Analysis of variance and significance check of regression equation for mean diameter of nanofibers.

Source of Variance	Square Sum	Mean Square	F-Statistic	*p*-Value	
Model	0.0864	0.0096	60.50	<0.0001	significant
A	0.0003	0.0003	0.9117	0.3715	not significant
B	0.0010	0.0010	6.31	0.0402	significant
C	0.0512	0.0512	325.85	<0.0001	significant
AB	0.0004	0.0004	2.87	0.1340	not significant
AC	0.0000	0.0000	0.0132	0.9119	not significant
BC	0.0000	0.0000	0.3849	0.5546	not significant
A^2^	0.0001	0.0001	0.1757	0.6877	not significant
B^2^	0.0002	0.0002	1.27	0.2970	not significant
C^2^	0.0330	0.0330	204.40	<0.0001	significant

## Data Availability

The original contributions presented in the study are included in the article, further inquiries can be directed to the corresponding author.
